# Digital Health Research Symposium: Closing Panel Commentary

**DOI:** 10.1016/j.mcpdig.2024.06.003

**Published:** 2024-09-17

**Authors:** Judd E. Hollander, Kristin L. Rising, Brian M. Dougan

**Affiliations:** Department of Emergency Medicine, Sidney Kimmel Medical College of Thomas Jefferson University, Philadelphia, PA; Division of General Internal Medicine, Mayo Clinic, Rochester, MN

### Scalability—Judd Hollander, MD

Those of us in the innovation space within health systems have had to evolve from embracing innovations that were cool to adopting innovations that have a positive return on investment. Health systems are under a tremendous financial crunch.

At the end of the day, I think scalability comes down to a single question—Is it better than what you do now? Or is it good enough and less costly? We may not need to do the best of everything, but there is a threshold we need to get over, and we need to accept the fact that we might not all be driving whatever you consider the most elite car, but we all need one that can reliably get us safely from one location to another. That is the way we need to think about digital health.

The abstract presentations were great. The presentation on validating a portable, camera-based system to scale the clinical gait assessment as a telehealth solution is great work. My iPhone 14 can detect falls; it can measure distance, speed, gait asymmetry, heart rate speed, steadiness, and step length. It also does something else—it uses the global positioning system and notifies someone where I am should I fall. I question if the whole study is about fall prevention, what technology could be more scalable than the iPhone or a watch? As I understand the presentation, the device that was put together in the clinic worked better than a more expensive device that was being used previously. The real question is does it work better than what many people already have? That would mean, in terms of scalability, you lose, because you are going to cost more money than an iPhone. Maybe it can work better? If it is better, then there's a case to be made for it.

I have questions regarding the abstract on transforming large language models (LLM) into superior clinical decision support tools by embedding clinical practice guidelines. I am involved a little bit with artificial intelligence (AI) and will share my concerns as health systems race to use it for specific items such as preauthorization, payers will also be racing to develop AI so that they could continue to deny just as many procedures. Are we just having an arms race using AI as the tool? The second is why do we each need to build our own LLM? Can we have one good health care LLM? These are very expensive to design so it is not going to be scalable or equitable for every hospital to develop their own LLM.

In terms of scalability, we need to be creative and work together even though our health systems may be competitive with each other, at times, around market share. It is great stuff and all of this is moving the field ahead.

### Patient Engagement—Kristin Rising, MD

Our Center’s work is firmly embedded in patient engagement from very early on in project conception, in which we regularly engage with patients and communities to determine what is broken about systems, all the way through to project completion. As my team has heard from many patients, a common barrier to digital health uptake is a lack to desire to change. We need to engage with patients before we roll out new services, partner with them from the beginning. Back to those opening comments in this webinar regarding what patient engagement is, for me the term “patient engagement” means working with patients as co-partners and co-designers.

Regarding the patient engagement presentations, both raised very interesting points and questions. The first talk assessed differences in how data are presented on device labels, with the background for this work being that there is a lack of data on how to communicate most effectively regarding AI-related devices. Their findings highlighted how differences in the types of data presented impacted patients’ trust and acceptability of the devices. A primary question I had for this work was how efficacy was defined, and whether patients had input in this decision. Although communication efficacy from a company viewpoint may well be best encapsulated as device uptake, I would think that communication efficacy from the patient viewpoint may align more with whether they had sufficient understanding regarding the device to make an informed decision about its uptake. My goal in working with patients to educate them about telemedicine is not to get everyone to use it. Rather, I want to ensure that everyone has the skills and knowledge needed to make informed decisions about whether they want to use it. Applying this approach to the presentation on device labeling, I would be interested in including assessments of how to get people most informed about what the device is.

Moving on to the study on colonoscopy uptake, this was a very exciting project focused on getting real time information to patients at a time that is useful, with the goal of increasing colonoscopy completion rates. I was very interested in receiving more information on how the program was developed. Specifically, was it developed with patients, and was user testing done before launch? Although digital health has the potential to improve health outcomes, there is a threat that it will increase health disparities if we are not intentional about ensuring uptake across the population. As discussed above, in addition to digital literacy, trust is a common barrier to telehealth uptake. A lot of patients do not trust getting their health information on their device, and thus I suspect they would be very hesitant to use this program. I am interested in whether patients were engaged to co-design this tool, and what the rate of uptake was among the patients approached. In this case, co-design would ideally include both patients who had had low-show rates for their colonoscopy and those who have low digital literacy and trust.

### Team Dynamics—Brian Dougan, MD

Digital health solutions are advancing quickly, requiring careful attention to selecting and applying digital solutions to impact team dynamics positively. Although applying digital solutions, we aim to meet patient needs while sustaining high-functioning teams as they advance care quality, safety, and patient experience. Selected digital solutions should be a value-add for our patients as well as the care team in an era when healthcare teams are addressing substantial challenges. Our teams need support addressing the needs of an aging population and managing large amounts of data in a more usable format. As we consider the impact of potential new digital solutions, we should ask whether the solution makes it easier for our team to do the right thing for our patients. Does the solution bring the team together or separate them into silos? Does the solution reduce work for our teams or add additional steps?

As we build our practices, sometimes the result is an efficient model for the team, though not always patient-centered.

Dr Fogelson’s presentation builds on the idea of expanding our neighborhood through emergency telehealth care for pediatric patients. This is a wonderful example of leveraging digital health to meet rural care needs. As a former rural primary care physician, I appreciate having team members available for challenging and complex care. I cannot imagine a more vital time to have expertise available than when serving pediatric emergency room patients. Her study found the significant value of telehealth in the emergency room setting in helping rural physicians address questions such as when a patient is safe to go home vs the need for hospitalization or how to most skillfully stabilize a pediatric trauma patient while awaiting transport to a higher level of care.

As we consider the digital health solutions available to us, not all the literature will be generalizable to every practice. There are ample opportunities to consider how digital health solutions will impact specific practices. In the setting of a significant primary care shortage and an innovation bias toward adding more steps to our work, we need to study digital solutions that may subtract workload, organize, and condense large amounts of patient data and allow teams to work more efficiently together. Additional areas that need to be studied include identifying digital solutions that reduce cost, improve patient experience and safety, and decrease care team burnout.

## Potential Competing Interests

The authors report no competing interests.

